# Risk Factors for West Nile Neuroinvasive Disease and Mortality in the US, 2013-2024

**DOI:** 10.1001/jamanetworkopen.2025.48229

**Published:** 2025-12-10

**Authors:** Seth D. Judson, David Dowdy

**Affiliations:** 1 Division of Infectious Diseases, Department of Medicine, David Geffen School of Medicine at University of California, Los Angeles; 2 Department of Epidemiology, Johns Hopkins Bloomberg School of Public Health, Baltimore, Maryland

## Abstract

**Importance:**

West Nile virus (WNV) is the leading mosquito-borne infection in the US, causing West Nile fever or West Nile neuroinvasive disease (WNND) with substantial morbidity and mortality. Contemporary analyses are needed to identify at-risk populations and to target interventions.

**Objective:**

To assess risk factors associated with WNND and mortality among adults with WNV infection using a national cohort from federated data.

**Design, Setting, and Participants:**

This retrospective cohort study of deidentified electronic medical record data from the TriNetX research network from January 2013 to December 2024 included patients from 65 health care organizations from across the US. Data were accessed from March 28 to April 30, 2025.

**Exposures:**

Demographic characteristics (age, sex, race, and ethnicity), comorbidities (hematologic malignant neoplasms, heart disease, diabetes, HIV infection, chronic kidney disease [CKD], liver disease, hypertension, alcohol-related disorders, cerebrovascular disease [CEVD], chronic obstructive pulmonary disease, asthma, multiple sclerosis, dementia, rheumatoid arthritis, and organ transplant), and medications (immune suppressants, antineoplastics).

**Main Outcomes and Measures:**

Primary outcomes were WNND development and all-cause mortality (at 30 days, 90 days, and overall).

**Results:**

Among the initial cohort of 3064 patients with a diagnosis of WNV infection (per the *International Statistical Classification of Diseases and Related Health Problems, Tenth Revision *codes), those with demographic data included 1249 (53%) who were consistent with West Nile fever (624 females [50%]; mean [SD] age, 55 [17] years) and 1127 (47%) who were consistent with WNND (691 males [61%]; mean [SD] age, 59 [17] years). Risk factors associated with WNND included age (adjusted hazard ratio [AHR], 1.10 [95% CI, 1.06-1.15] per decade), male sex (AHR, 1.29 [95% CI, 1.15-1.45]), CKD (AHR, 1.21 [95% CI, 1.00-1.45]), CEVD (AHR, 1.22 [95% CI, 1.03-1.45]), hematologic malignant neoplasms (AHR, 1.38 [95% CI, 1.09-1.76]), immune suppressant use (AHR, 1.43 [95% CI, 1.11-1.83]), hypertension (AHR, 1.18 [95% CI, 1.04-1.34]), alcohol-related disorders (AHR, 1.54 [95% CI, 1.20-1.97]), and multiple sclerosis (AHR, 2.34 [95% CI, 1.62-3.37]). Significant risk factors associated with mortality were WNND (AHR, 2.49 [95% CI, 1.37-4.52] for 30-day mortality), age (AHR, 1.32 [95% CI, 1.07-1.60] per decade), CKD (AHR, 2.08 [95% CI, 1.01-3.93]), and CEVD (AHR, 2.00 [95% CI, 1.14-3.50]).

**Conclusions and Relevance:**

In this cohort study of patients with WNV infection, multiple comorbidities were identified as risk factors associated with WNND; and patients who developed WNND had increased mortality risk. Targeted prevention strategies and countermeasures for high-risk individuals may substantially reduce morbidity and mortality.

## Key Points

**Question:**

What were the risk factors associated with West Nile neuroinvasive disease and mortality in the US between 2013 and 2024?

**Findings:**

In this cohort study of 3064 adults, older age, chronic kidney disease, and cerebrovascular disease were significantly associated with both West Nile neuroinvasive disease and mortality. Additionally, male sex, hematologic malignant neoplasms, immune suppressants, hypertension, alcohol-related disorders, and multiple sclerosis were risk factors significantly associated with West Nile neuroinvasive disease.

**Meaning:**

The findings of this study suggest that an aging population in the US, with a rising prevalence of comorbidities and immunosuppression, may be increasingly at higher risk of severe disease from West Nile virus infection.

## Teaser

This cohort study assesses the risk factors associated with West Nile neuroinvasive disease and mortality in the US between 2013 and 2024.

## Introduction

West Nile virus (WNV) first emerged in the US in 1999 and is an ongoing threat, causing more than 80% of all cases of mosquito-borne infections in the US annually.^1^ Now endemic throughout most of the continental US, WNV is transmitted from bird reservoir hosts to humans primarily via *Culex* mosquitoes.^2^ It is estimated that approximately 75% of people infected with WNV are asymptomatic, while 25% develop a nonspecific febrile syndrome called West Nile fever (WNF).^2,3,4^ A subset of WNV infections (<1%) can progress to West Nile neuroinvasive disease (WNND), which has a case fatality of approximately 10% and can cause lifelong neurological complications.^5,6^

One of the priorities for the 2024 US Centers for Disease Control and Prevention (CDC) National Public Health Strategy to Prevent and Control Vector-Borne Diseases in People is to reduce the annual number of WNND cases to below 500 by 2035.^1^ To achieve this goal, a better understanding is needed of the risk factors associated with WNND and mortality. Unfortunately, the majority of risk factor analyses for WNND in the US were performed over a decade ago and are limited in geographic and/or population scope.^7,8,9^ One of the largest risk factor analyses covering multiple regions in the US was a retrospective cohort from 2008 to 2010 using CDC ArboNET data from 19 jurisdictions from primarily western and midwestern states and included 1090 adult WNV cases (641 with WNND).^8^ Additionally, comparisons of risk factors have been limited by study heterogeneity and small sample sizes, as noted by both a scoping review and a recent systematic review and meta-analysis.^7,9^ Given changing demographics and increasing prevalence of comorbidities, such as immunosuppression, in the US, there is a need to reassess risk factors for WNND and disease severity.^10^

Recent advancements in research networks and electronic medical records over the past decade provide new opportunities to analyze additional data in the context of WNV infection. In particular, federated learning provides the opportunity to analyze infectious disease data from multiple clinical sources,^11^ substantially increasing sample sizes and geographic representation. To address gaps in our existing knowledge regarding risk factors associated with WNV infection, we used the TriNetX research network,^12^ a federated data and analytics platform from a large research network of health care organizations (HCOs), to identify factors associated with WNND and mortality across the US from January 2013 to December 2024.

## Methods

### Ethical Considerations

This retrospective cohort study examined data from the TriNetX research network and was determined to be exempt from institutional review board review by the Johns Hopkins School of Medicine institutional review board, as it included only secondary analyses of existing aggregate data that are deidentified per the standard defined in Section §164.514(a) of the HIPAA (US Health Insurance Portability and Accountability Act) privacy rule. The requirement for informed consent was therefore waived. The study followed the Strengthening the Reporting of Observational Studies in Epidemiology (STROBE) cohort checklist for reporting guideline.

### Cohort Identification

We identified retrospective cohorts from querying the TriNetX research network, a federated data and analytics platform containing data from more than 100 HCOs across the US. Using *International Classification of Diseases and Related Health Problems, Tenth Revision *(*ICD-10*) codes, we formed multiple cohorts of deidentified and aggregated data from adult patients (≥18 years of age) diagnosed with WNV infection from 2013 to 2024 (eTable 1 in Supplement 1). We used standardized *ICD-10* codes for mutually exclusive groups of WNF (A92.30, A92.39) and WNND (A92.31, A92.32). Data from the TriNetX research network were accessed from March 28 to April 30, 2025. We also compared the patient counts with national CDC ArboNET data from 2013 to 2024.^13^

### Risk Factor Identification

**Figure 1.**
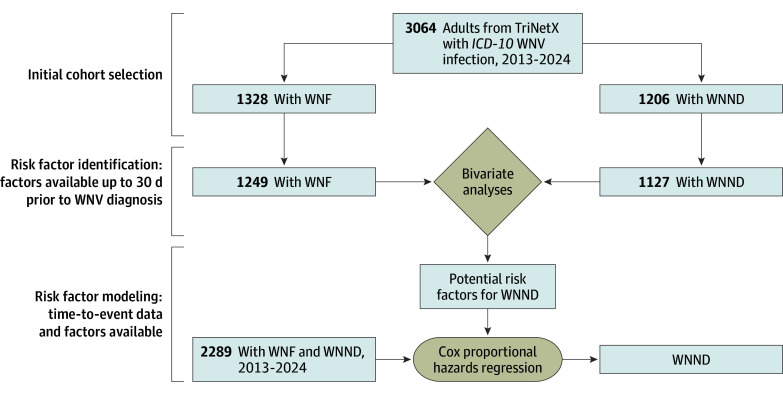
Cohort Selection and Analytic Framework for West Nile Neuroinvasive Disease (WNND) The flow diagram depicts the cohort selection and analysis process in TriNetX. The initial cohort included all patients with *International Statistical Classification of Diseases and Related Health Problems, Tenth Revision *(*ICD-10*) codes for West Nile virus (WNV) infection (with subgroups of West Nile fever [WNF] and WNND). Those patients with data available within a 30-day period before or on the same day as the WNV infection diagnosis were included in the bivariate analyses. Significant factors from the bivariate analyses and patients with time-to-event data available were then included in the multivariable Cox proportional hazards regression model with the outcome of WNND.

**Figure 2.**
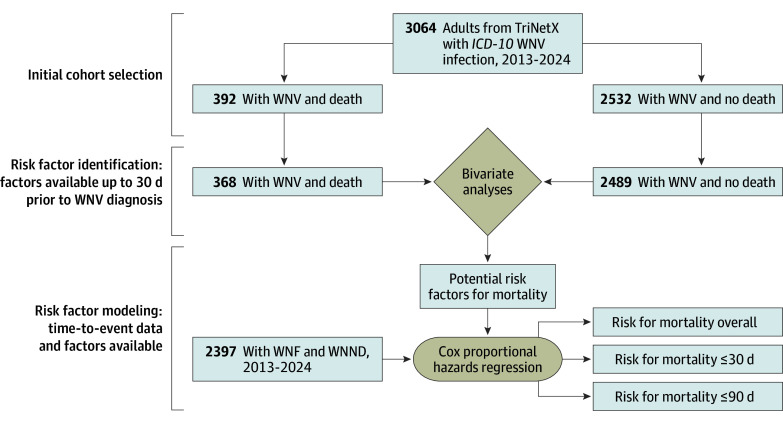
Cohort Selection and Analytic Framework for West Nile Virus (WNV) Infection Mortality The flow diagram depicts the cohort selection and analysis process in TriNetX. The initial cohort included all patients with *International Statistical Classification of Diseases and Related Health Problems, Tenth Revision *(*ICD-10*) codes for WNV infection (with subgroups of subsequent death or no death). Those patients with data available within a 30-day period before or on the same day as the WNV infection diagnosis were included in the bivariate analyses. Significant factors from the bivariate analyses and patients with time-to-event data available were then included in multivariable Cox proportional hazards regression models with the outcomes of mortality overall, mortality within 30 days, and mortality within 90 days. WNF indicates West Nile fever; WNND, West Nile neuroinvasive disease.

To identify potential risk factors associated with WNND and mortality, we conducted a literature review in PubMed using the search terms *West Nile* and *risk*. We also referenced a scoping review from 2017 and a systematic review and meta-analysis from 2025 to identify demographic and clinical risk factors that have been studied in association with WNV infection outcomes.^7,9^ We selected risk factors that previously have been assessed or associated with WNND, severity, and/or mortality. Our final list of potential risk factors included age, sex, race, ethnicity, 16 comorbidities, and 5 classes of medications (eTable 2 in Supplement 1). For our analysis, we included risk factors that were present within a 30-day period before or on the same day as the WNV diagnosis (details are provided in the eMethods in Supplement 1). Flow diagrams for cohort selection and analysis are shown in Figure 1 and Figure 2. Race and ethnicity have been previously studied as risk factors associated with WNND and WNV mortality^7^ and were thus included in the study. The categories were defined by the TriNetX research network for race (American Indian or Alaska Native, Asian, Black or African American, Native Hawaiian or Other Pacific Islander, White, other [patients with race not matching the other categories], and unknown) and for ethnicity (Hispanic or Latino, non-Hispanic or non-Latino, and unknown).

### Statistical Analysis

Descriptive statistics of the TriNetX cohorts were calculated in TriNetX, which compares continuous data using independent *t* tests and categorical data using χ^2^ or Fisher exact tests. We selected potential risk factors that were significant in bivariate analyses (2-sided *P* < .05) to use in our multivariable Cox proportional hazards regression analyses, with the exceptions of subgroups of immune suppressants (tacrolimus and mycophenolate mofetil [MMF]) and solid organ transplant, which both had a high level of overlap with the broader category of immune suppressant use.

The primary outcomes for our analysis were WNND and all-cause mortality. Given that the analytic functionality of TriNetX is geared toward a time-to-event analysis, we used multivariable Cox proportional hazards regression models for our primary analyses. We explored risk factors associated with developing WNND and for mortality among those diagnosed with either WNND or WNF (considering WNND as a risk factor for mortality). As mortality associated with WNV may vary by time period, we compared overall mortality as well as acute mortality (within 30 days and within 90 days). TriNetX lacks functionality to test the proportionality assumption in multivariable models but can test this assumption in univariable analysis with categorical covariates. Therefore, we checked the proportionality assumption through 2 mechanisms. First, we conducted a univariable analysis with each categorial covariate that was significantly associated with WNND and used Schoenfeld residuals to evaluate proportionality (performed in TriNetX using a χ^2^ test; *P* > .05 suggests the proportionality assumption likely holds for that covariate). For mortality, we also compared adjusted hazard ratios (AHRs) across different time segments (overall, 30 days, and 90 days). Statistical analyses were performed on the TriNetX platform.

As a federated data platform, TriNetX queries data from the electronic medical record systems of each HCO, which have been pseudonymized and transferred to a server behind each HCO’s firewall.^14^ The TriNetX algorithms are run on these data, and the summary results are returned. To maintain anonymity of the data, the specific HCO notifying each case is not identified. As such, neither individual AHRs for the HCOs nor AHRs accounting for clustering by HCO can be reported.

## Results

### Characteristics of Patients With WNV

Overall 3064 adults were diagnosed with WNV infection from 65 HCOs in TriNetX from 2013 to 2024; those with demographic data included 1249 (53%) with WNF (624 females [50%], 594 males [48%], and 31 with unknown sex [2%]; ≤10 American Indian or Alaska Native [1%], 29 Asian [2%], 75 Black or African American [6%], ≤10 Native Hawaiian or Other Pacific Islander [1%], 58 other race [5%], 115 unknown race [9%], and 963 White [77%]; 90 Hispanic or Latino [7%], 977 non-Hispanic or non-Latino [78%], and 182 unknown ethnicity [15%]; mean [SD] age, 55 [17] years) and 1127 (47%) with WNND (425 females [38%], 691 males [61%], and 11 with unknown sex [1%]; ≤10 American Indian or Alaska Native [1%], 28 Asian [2%], 102 Black or African American [9%], ≤10 Native Hawaiian or Other Pacific Islander [1%], 52 other race [5%], 84 unknown race [7%], and 849 White [75%]; 90 Hispanic or Latino [8%], 843 non-Hispanic or non-Latino [75%], and 194 unknown ethnicity [17%]; mean [SD] age, 59 [17] years).

The distribution of the 3064 patients by geographic region included 1053 (35%) in the West, 1052 (35%) in the South, 459 (15%) in the Midwest, and 379 (13%) in the Northeast. Baseline characteristics of the cohorts stratified by WNND are shown in Table 1, and characteristics by mortality are presented in eTable 3 in Supplement 1. Compared with CDC ArboNET data, the TriNetX cohort represented 13% (3064 of 23 904) of nationally reported WNV infection cases and 8% (1206 of 15 610) of nationally reported WNND cases from 2013 through 2024.

**Table 1.** Baseline Characteristics of Patients With WNND and WNF in the US From 2013 to 2024

Covariate	Patients, No. (%)		*P* value^a^
	WNND (n = 1127)	WNF (n = 1249)	
Age at diagnosis, mean (SD), y	59 (17)	55 (17)	<.001
Sex			
Female	425 (38)	624 (50)	<.001
Male	691 (61)	594 (48)	<.001
Unknown	11 (1)	31 (2)	.005
Ethnicity			
Hispanic or Latino	90 (8)	90 (7)	.47
Non-Hispanic or non-Latino	843 (75)	977 (78)	.05
Unknown	194 (17)	182 (15)	.08
Race			
American Indian or Alaska Native	≤10 (1)^b^	≤10 (1)^b^	.82
Asian	28 (2)	29 (2)	.80
Black or African American	102 (9)	75 (6)	.005
Native Hawaiian or Other Pacific Islander	≤10 (1)^b^	≤10 (1)^b^	.82
Other^c^	52 (5)	58 (5)	.97
Unknown	84 (7)	115 (9)	.12
White	849 (75)	963 (77)	.31
Comorbidity			
Hematologic malignant neoplasms	54 (5)	24 (2)	<.001
Ischemic heart diseases	140 (12)	91 (7)	<.001
Other forms of heart disease	285 (25)	202 (16)	<.001
Diabetes	218 (19)	160 (13)	<.001
HIV infection	12 (1)	12 (1)	.80
Chronic kidney disease	122 (11)	77 (7)	<.001
Diseases of the liver	66 (6)	74 (6)	.94
Essential (primary) hypertension	420 (37)	329 (26)	<.001
Alcohol-related disorders	57 (5)	25 (2)	<.001
Cerebrovascular diseases	155 (14)	78 (6)	<.001
Chronic obstructive pulmonary disease	58 (5)	33 (3)	.002
Asthma	44 (4)	65 (5)	.13
Multiple sclerosis	23 (2)	≤10 (1)^b^	.01
Dementia	17 (2)	19 (2)	.98
Other rheumatoid arthritis	25 (2)	19 (2)	.21
Transplanted organ	38 (3)	19 (2)	.003
Medication			
Immune suppressants (overall)	52 (5)	25 (2)	<.001
Tacrolimus	28 (2)	12 (1)	.004
Mycophenolate mofetil	31 (3)	≤10 (1)^b^	<.001
Antineoplastics	23 (2)	31 (2)	.47
Prednisone	71 (6)	222 (7)	.73

Abbreviations: WNF, West Nile fever; WNND, West Nile neuroinvasive disease.

^a^ Continuous data were calculated using independent *t* tests, and categorical data were calculated using χ^2^ or Fisher exact tests.

^b^ TriNetX patient counts of 10 or fewer were rounded to 10 to maintain anonymity.

^c^ Included patients with race not matching the other categories.

### Risk Factors Associated With WNND

Patient-level characteristics that were significantly associated with WNND in bivariate analyses included age; male sex; Black or African American race; ischemic heart disease; other heart disease; malignant neoplasms of lymphoid, hematopoietic, and related tissue (hematologic malignant neoplasms); diabetes; chronic kidney disease (CKD); hypertension; alcohol-related disorders; chronic obstructive pulmonary disease; cerebrovascular disease (CEVD); multiple sclerosis (MS); solid organ transplant; and immune suppressant use (including subgroups of tacrolimus and MMF).

In multivariable Cox proportional hazards regression analyses, risk factors significantly associated with WNND included age (per decade) (AHR, 1.10 [95% CI, 1.06-1.15]), male sex (AHR, 1.29 [95% CI, 1.15-1.45]), CKD (AHR, 1.21 [95% CI, 1.00-1.45]), CEVD (AHR, 1.22 [95% CI, 1.03-1.45]), hematologic malignant neoplasms (AHR, 1.38 [95% CI, 1.09-1.76]), immune suppressant use (AHR, 1.43 [95% CI, 1.11-1.83]), hypertension (AHR, 1.18 [95% CI, 1.04-1.34]), alcohol-related disorders (AHR, 1.54 [95% CI, 1.20-1.97]), and MS (AHR, 2.34 [95% CI, 1.62-3.37]) (Table 2 and eFigure 1 in Supplement 1). The proportionality assumption was met for sex (χ^2^ = 0.41; *P* = .52), CKD (χ^2^ = 0.33; *P* = .56), CEVD (χ^2^ = 0.26; *P* = .61), hematologic malignant neoplasms (χ^2^ = 2.8; *P* = .10), immune suppressant use (χ^2^ = 0.09; *P* = .77), hypertension (χ^2^ = 0.22; *P* = .64), alcohol-related disorders (χ^2^ = 0.17; *P* = .68), and MS (χ^2^ = 2.4; *P* = .12).

**Table 2.** Time-to-Event Analysis for West Nile Neuroinvasive Disease

Covariate	AHR (95% CI)	*P* value
Male	1.29 (1.15-1.45)	<.001
Age (per 10 y)	1.10 (1.06-1.15)	<.001
Ischemic heart diseases	1.00 (0.84-1.19)	>.99
Other forms of heart disease	1.14 (0.99-1.32)	.07
Chronic kidney disease	1.21 (1.00-1.45)	.047
Cerebrovascular diseases	1.22 (1.03-1.45)	.02
Chronic obstructive pulmonary disease	1.09 (0.78-1.51)	.62
Diabetes	1.02 (0.87-1.18)	.84
Hematologic malignant neoplasms	1.38 (1.09-1.76)	.008
Black or African American race	1.18 (0.96-1.45)	.12
Diseases of the liver	0.85 (0.68-1.06)	.14
Non-Hispanic or non-Latino ethnicity	0.95 (0.84-1.07)	.39
Immune suppressants	1.43 (1.11-1.83)	.005
Essential (primary) hypertension	1.18 (1.04-1.34)	.008
Alcohol-related disorders	1.54 (1.20-1.97)	.001
Multiple sclerosis	2.34 (1.62-3.37)	<.001

Abbreviation: AHR, adjusted hazard ratio.

### Risk Factors Associated With Mortality

Overall, 392 of 3064 patients with WNV infection (13%), from 2013 through 2024, died; of those deaths, 129 (33%) occurred within 30 days of WNV infection diagnosis. Among those who died, 197 (50%) had a prior diagnosis of WNND (*ICD-10* codes A92.31 and A92.32), 110 (28%) had WNF (*ICD-10 *codes A92.30 and A92.39), and 85 (22%) had an unspecified WNV infection (*ICD-10 *code A92.3). The overall case fatality rate for WNND in this cohort was 16% (197 of 1206).

Potential risk factors associated with mortality in the TriNetX cohort that were significant in the bivariate analyses included age, male sex, non-Hispanic or non-Latino ethnicity, Black or African American race, ischemic heart disease, other heart disease, hematologic malignant neoplasms, diabetes, HIV infection, CKD, liver disease, chronic obstructive pulmonary disease, CEVD, dementia, solid organ transplant, immune suppressants (including subgroups of tacrolimus and MMF), prednisone, and antineoplastics.

In multivariable Cox proportional hazards regression analyses for mortality, several factors were identified as significant across all time frames (30 days, 90 days, and overall). For 30-day mortality, these included WNND (AHR, 2.49 [95% CI, 1.37-4.52]), age (per decade) (AHR, 1.32 [95% CI, 1.07-1.60]), other heart disease (AHR, 5.50 [95% CI, 2.96-10.23]), CKD (AHR, 2.08 [95% CI, 1.01-3.93]), and CEVD (AHR, 2.00 [95% CI, 1.14-3.50]) (Table 3 and eFigure 2 in Supplement 1). The results for 90-day mortality are available in eTable 4 and eFigure 3 in Supplement 1 and for overall mortality in eTable 5 and eFigure 4 in Supplement 1. The AHRs for WNND, CKD, CEVD, and age were proportional across time windows, while the AHR for other heart disease was significantly higher within 30 days. The proportionality assumption was met for WNND (χ^2^ = 0.002; *P* = .96), CKD (χ^2^ = 0.59; *P* = .44), and CEVD (χ^2^ = 1.30; *P* = .26).

**Table 3.** Time-to-Event Analysis for Mortality Within 30 d of West Nile Virus Infection

Covariate	AHR (95% CI)	*P* value
WNND	2.49 (1.37-4.52)	.003
Male sex	0.65 (0.39-1.08)	.01
Age (per 10 y)	1.32 (1.07-1.60)	.008
Ischemic heart diseases	1.07 (0.57-2.01)	.82
Other forms of heart disease	5.50 (2.96-10.23)	<.001
Chronic kidney disease	2.08 (1.01-3.93)	.02
Cerebrovascular diseases	2.00 (1.14-3.50)	.02
Chronic obstructive pulmonary disease	1.50 (0.66-3.42)	.33
Diabetes	0.59 (0.31-1.15)	.11
Hematologic malignant neoplasms	1.55 (0.60-4.04)	.37
Black or African American race	0.68 (0.22-2.09)	.50
Immune suppressants	0.58 (0.17-2.04)	.40
Unspecified dementia	0.43 (0.06-3.21)	.41
Diseases of the liver	1.95 (0.93-4.08)	.08
HIV infection	0.57 (0.06-5.31)	.62
Antineoplastics	0.72 (0.09-5.41)	.75
Prednisone	1.22 (0.45-3.32)	.70
Non-Hispanic or non-Latino ethnicity	0.95 (0.51-1.78)	.88

Abbreviations: AHR, adjusted hazard ratio; WNND, West Nile neuroinvasive disease.

## Discussion

In this cohort study using federated data, we analyzed a large national cohort of patients diagnosed with WNV infection. Our analysis, which to our knowledge is the most extensive contemporary US study of WNND and mortality risk factors (more than 2000 patients from 65 HCOs between 2013 and 2024), corroborates previous evidence with a larger sample size and more granularity. Specifically, we identified older age, CKD, and CEVD as risk factors associated with both WNND and mortality (across all time frames), and male sex, hematologic malignant neoplasms, immune suppressant use, hypertension, alcohol-related disorders, and MS as risk factors associated with WNND.

Our findings are consistent with prior consensus that older age is associated with WNND and mortality,^7,8^ and male sex has been identified as a risk factor for WNND in approximately half of the studies.^7^ Clinical characteristics associated with WNND and mortality in prior studies have varied. A recent review and meta-analysis identified hypertension, cancer, and diabetes as significantly associated with WNND, while CKD was associated with mortality.^9^ A limited number of studies have identified cardiovascular disease, liver disease, alcohol use, and autoimmune disease as risk factors associated with WNND and mortality.^7,9^ We identified similar conditions as significant risk factors associated with WNND; diabetes and liver disease were exceptions, possibly because we did not stratify for disease severity.

Immunocompromising conditions and immunosuppression are also widely considered to be risk factors associated with WNV disease severity, although most prior analyses have been unable to distinguish between specific conditions and medications owing to sample-size limitations.^7,8^ A recent retrospective cohort analysis of patients from the Mayo Clinic found that immunosuppression is associated with severity of clinical manifestations of WNND.^10^ We similarly found that the risk for WNND was increased for specific immunocompromising conditions (hematologic malignant neoplasms and MS) and for those receiving immune suppressants (including solid organ transplant recipients).

These risk factors appear to align with our current understanding of the pathogenesis of WNND. Multiple components of the immune system (including innate, humoral, and cellular immunity) are involved in mitigating WNV infection.^15^ Additionally, WNV enters the central nervous system (CNS) through multiple pathways, one of which includes permeability of the blood-brain barrier.^6,16^ Conditions that disrupt the immune system or cause increased CNS risk through permeability of the blood-brain barrier or inflammation may lead to increased risk of WNND and sequelae.^15,16^ The risk factors identified in this study associated with WNND and mortality broadly fit into these 2 categories: conditions that specifically impair the immune system (hematologic malignant neoplasms and immune suppressant use) and conditions that increase CNS risk (hypertension, heart disease, and CEVD). Age, sex, CKD, and MS may contribute to both immune impairment and CNS risk. We emphasize that this framework is not an established mechanism but rather a hypothesis-generating model to guide further research. Further mechanistic and causal inference studies are needed to elucidate the causal pathways by which these risk factors may contribute to WNND and mortality.

Given that there is no effective treatment or licensed vaccine for WNV in humans, it is essential to identify populations that are at increased risk for WNND and mortality. Our findings could inform clinical prediction tools for triaging patients diagnosed with WNV infection. For instance, a risk score could help guide whether a patient with WNV should be admitted for closer monitoring. These analyses could also assist ongoing efforts by public health officials to model and forecast WNND for targeted interventions.^17^ If a WNV vaccine becomes available, for example, a targeted vaccination strategy among those at highest risk for WNND could be cost-effective and impactful.^18^ While existing WNV vaccine cost-effectiveness analyses have focused on age-based strategies,^19^ a strategy including additional risk factors, such as those identified in this study, may be even more cost-effective and feasible (eg, by centering vaccine campaigns in clinics treating these conditions).

Overall, we found a greater than 2-fold risk of death associated with WNND, adjusting for other comorbidities. This risk, coupled with the increasing prevalence of multiple risk factors associated with WNND (such as age, immunocompromise, hypertension, heart disease, CEVD, and CKD), indicates that a growing population in the US may be at risk for morbidity and mortality following WNV infection.

Our approach of using federated data from TriNetX allowed us to compare multiple characteristics and risk factors across a large cohort. Our study design addressed prior challenges in risk factor analyses for WNV infection outcomes by including: (1) larger sample sizes to compare multiple risk factors,^8,20^ (2) longer cohort follow-up, given that acute and convalescent phase mortality from WNV may differ,^21^ and (3) broader geographic representation.

### Limitations

Key limitations to this study include that the cases are based on *ICD-10* codes, which may be subject to misclassification and affect risk estimates. Further studies of laboratory-confirmed cases and use of functional outcome measures are needed to assess the validity of *ICD-10* codes for WNV infection. There is also no current functionality on the TriNetX platform to perform collinearity diagnostics, evaluate clustering by HCO, or model time-varying effects, and several covariates (such as CEVD and heart disease) may be correlated and/or have dynamic effects on risk. Our estimates of variance may therefore be overly narrow, as they do not account for such residual uncertainty. Likewise, conclusions regarding causality cannot be made, given the possibility of reverse causality. For instance, rarely has WNV been associated with acute cerebrovascular events, which could be categorized as CEVD in TriNetX.^22^ Additionally, academic medical centers and acute care settings form a large part of the TriNetX network; our results may therefore have less generalizability to lower-acuity clinical settings.^23^ Federated data networks such as TriNetX provide the opportunity for analyzing deidentified patient data from multiple regions, providing more statistical power. Nevertheless, local cohorts remain important for further analyzing these observations and exploring specific nuances through access to individual-level data.

## Conclusions

In this cohort study of patients with an *ICD-10* diagnosis of WNV infection, multiple comorbidities were associated with WNND, which could broadly be categorized by impaired immunity and CNS risk. Mortality was associated with WNND, increased age, CEVD, and CKD. As the population in the US and many countries continues to age with increasing comorbidities, it will be essential to continue to identify and respond to the growing risk of morbidity and mortality associated with WNV.

## Notes

Supplement 1.
eMethods.
**eTable 1. **TriNetX Cohorts **eTable 2. **Initial Cohort Characteristics Assessed **eTable 3. **Baseline Characteristics of WNV and Death vs WNV and No Death 2013-2024 Cohorts **eTable 4. **Mortality Within 90 days **eTable 5. **Overall Mortality **eFigure 1. **Time-to-Event Analysis: WNND **eFigure 2. **Time-to-Event Analysis: 30-Day Mortality **eFigure 3. **Time-to-Event Analysis: 90-Day Mortality **eFigure 4. **Time-to-Event Analysis: Overall Mortality

Supplement 2.
Data Sharing Statement

## References

[1] Vector-borne diseases: National Public Health Strategy to Prevent and Control Vector-Borne Diseases in People. US Centers for Disease Control and Prevention. February 13, 2025. Accessed October 29, 2025. https://www.cdc.gov/vector-borne-diseases/php/data-research/national-strategy/index.html

[2] Petersen LR. Epidemiology of West Nile virus in the United States: implications for arbovirology and public health. J Med Entomol. 2019;56(6):1456-1462. doi:10.1093/jme/tjz085 31549728

[3] Zou S, Foster GA, Dodd RY, Petersen LR, Stramer SL. West Nile fever characteristics among viremic persons identified through blood donor screening. J Infect Dis. 2010;202(9):1354-1361. doi:10.1086/656602 20874087

[4] Sejvar JJ. The long-term outcomes of human West Nile virus infection. Clin Infect Dis. 2007;44(12):1617-1624. doi:10.1086/518281 17516407

[5] McDonald E, Mathis S, Martin SW, Staples JE, Fischer M, Lindsey NP. Surveillance for West Nile virus disease—United States, 2009-2018. MMWR Surveill Summ. 2021;70(1):1-15. doi:10.15585/mmwr.ss7001a1 33661868 PMC7949089

[6] Fulton CDM, Beasley DWC, Bente DA, Dineley KT. Long-term, West Nile virus-induced neurological changes: a comparison of patients and rodent models. Brain Behav Immun Health. 2020;7:100105. doi:10.1016/j.bbih.2020.100105 34589866 PMC8474605

[7] Yeung MW, Shing E, Nelder M, Sander B. Epidemiologic and clinical parameters of West Nile virus infections in humans: a scoping review. BMC Infect Dis. 2017;17(1):609. doi:10.1186/s12879-017-2637-9 28877682 PMC5588625

[8] Lindsey NP, Staples JE, Lehman JA, Fischer M. Medical risk factors for severe West Nile virus disease, United States, 2008-2010. Am J Trop Med Hyg. 2012;87(1):179-184. doi:10.4269/ajtmh.2012.12-0113 22764311 PMC3391046

[9] Roberts JA, Kim CY, Hwang SA, . Clinical, prognostic, and longitudinal functional and neuropsychological features of West Nile virus neuroinvasive disease in the United States: a systematic review and meta-analysis. Ann Neurol. 2025;98(1):93-106. doi:10.1002/ana.27220 40008684

[10] Mbonde AA, Gritsch D, Harahsheh EY, . Neuroinvasive West Nile virus infection in immunosuppressed and immunocompetent adults. JAMA Netw Open. 2024;7(3):e244294. doi:10.1001/jamanetworkopen.2024.4294 38546642 PMC10979308

[11] Zwiers LC, Grobbee DE, Uijl A, Ong DSY. Federated learning as a smart tool for research on infectious diseases. BMC Infect Dis. 2024;24(1):1327. doi:10.1186/s12879-024-10230-5 39573994 PMC11580691

[12] Palchuk MB, London JW, Perez-Rey D, . A global federated real-world data and analytics platform for research. JAMIA Open. 2023;6(2):ooad035. doi:10.1093/jamiaopen/ooad035 37193038 PMC10182857

[13] West Nile virus: historic data (1999-2024). US Centers for Disease Control and Prevention. June 10, 2025. Accessed October 29, 2025. https://www.cdc.gov/west-nile-virus/data-maps/historic-data.html

[14] Ludwig RJ, Anson M, Zirpel H, . A comprehensive review of methodologies and application to use the real-world data and analytics platform TriNetX. Front Pharmacol. 2025;16:1516126. doi:10.3389/fphar.2025.1516126 40129946 PMC11931024

[15] Debiasi RL. West Nile virus neuroinvasive disease. Curr Infect Dis Rep. 2011;13(4):350-359. doi:10.1007/s11908-011-0193-9 21544522

[16] Kumar M, Nerurkar VR. In vitro and in vivo blood-brain barrier models to study West Nile virus pathogenesis. Methods Mol Biol. 2016;1435:103-113. doi:10.1007/978-1-4939-3670-0_9 27188553 PMC5502104

[17] Holcomb KM, Mathis S, Staples JE, . Evaluation of an open forecasting challenge to assess skill of West Nile virus neuroinvasive disease prediction. Parasit Vectors. 2023;16(1):11. doi:10.1186/s13071-022-05630-y 36635782 PMC9834680

[18] Gould CV, Staples JE, Huang CYH, Brault AC, Nett RJ. Combating West Nile virus disease—time to revisit vaccination. N Engl J Med. 2023;388(18):1633-1636. doi:10.1056/NEJMp2301816 37125778 PMC11627013

[19] Curren EJ, Shankar MB, Fischer M, Meltzer MI, Erin Staples J, Gould CV. Cost-effectiveness and impact of a targeted age- and incidence-based West Nile virus vaccine strategy. Clin Infect Dis. 2021;73(9):1565-1570. doi:10.1093/cid/ciab540 34117746 PMC9070563

[20] Sutinen J, Fell DB, Sander B, Kulkarni MA. Comorbid conditions as risk factors for West Nile neuroinvasive disease in Ontario, Canada: a population-based cohort study. Epidemiol Infect. 2022;150:e103. doi:10.1017/S0950268822000887 35543409 PMC9171902

[21] Philpott DCE, Nolan MS, Evert N, . Acute and delayed deaths after West Nile virus infection, Texas, USA, 2002-2012. Emerg Infect Dis. 2019;25(2):256-264. doi:10.3201/eid2502.181250 30667356 PMC6346437

[22] Zafar S, Dash D, Chachere M, Cowart J, Kass J. West Nile virus infection associated with central nervous system vasculitis and strokes (P03.264). *Neurology*. 2012;78(suppl 1):P03.264

[23] Nassar M, Abosheaishaa H, Elfert K, . TriNetX and real-world evidence: a critical review of its strengths, limitations, and bias considerations in clinical research. ASIDE Intern Med. 2025;1(2):24-33. doi:10.71079/ASIDE.IM.03222516 40697879 PMC12282508

