# Supplementary material for: Risk Factors for West Nile Neuroinvasive Disease and Mortality in the US, 2013-2024
Source: JAMA Netw Open. 2025 Dec 10;8(12):e2548229. doi: 10.1001/jamanetworkopen.2025.48229 (PMC12696598; doi:10.1001/jamanetworkopen.2025.48229)
Supplement: Supplement 1. — eMethods. eTable 1. TriNetX Cohorts eTable 2. Initial Cohort Characteristics Assessed eTable 3. Baseline Characteristics of WNV and Death vs WNV and No Death 2013-2024 Cohorts eTable 4. Mortality Within 90 days eTable 5. Overall Mortality eFigure 1. Time-to-Event Analysis: WNND eFigure 2. Time-to-Event Analysis: 30-Day Mortality eFigure 3. Time-to-Event Analysis: 90-Day Mortality eFigure 4. Time-to-Event Analysis: Overall Mortality [file jamanetwopen-e2548229-s001.pdf]

Supplemental Online Content

Judson SD, Dowdy D. Risk factors for West Nile neuroinvasive disease and mortality in the US. *JAMA Netw Open*. 2025;8(12):e2548229.
doi:10.1001/jamanetworkopen.2025.48229

eMethods
eTable 1. TriNetX Cohorts
eTable 2. Initial Cohort Characteristics Assessed
eTable 3. Baseline Characteristics of WNV and Death vs WNV and No Death 2013-2024 Cohorts
eTable 4. Mortality Within 90 days
eTable 5. Overall Mortality
eFigure 1. Time-to-Event Analysis: WNND
eFigure 2. Time-to-Event Analysis: 30-Day Mortality
eFigure 3. Time-to-Event Analysis: 90-Day Mortality
eFigure 4. Time-to-Event Analysis: Overall Mortality

This supplemental material has been provided by the authors to give readers additional information about their work.

© 2025 Judson SD, Dowdy D. *JAMA Network Open*.

eMethods

Risk factor selection

We first compared baseline characteristics and statistics between cohorts stratified by WNND and mortality. We selected characteristics based on risk factors that previously have been assessed or associated with WNND, severity, and/or mortality from literature review. Among those risk factors, we included those with sample size >10 individuals (which is the minimum sample size number that TriNetX reports to maintain anonymization). To distinguish among categories of immunosuppression, we separated HIV, solid organ transplant, and immune suppressant medications into separate categories. For cancer we specified hematologic malignancies, as this was the largest ICD-10 group in TriNetX that did not include benign neoplasms.

Our time window for risk factors included up to 30 days prior to and including the day of the index event (diagnosis of WNV infection). We selected among risk factors that were significant in bivariate analysis ($p < 0.05$) to use for the multivariable analyses.

Multivariable analysis

We compared the association between selected risk factors that were statistically significant in the bivariate analysis ($p < 0.05$ in S Table 3) in multivariable Cox proportional hazards models. Covariates were measured within 30 days prior to and including the day of the index event (diagnosis of WNV infection). Mortality was assessed at 30 days, 90 days, and any time after the index event. For the outcome of WNND, this was assessed any time after the index event, and the cohort included individuals with a healthcare visit in 2013-2024 prior to WNV diagnosis. Given the overlap between immune suppressant use and solid organ transplant, we selected immune suppressant use as a covariate for our final models given the larger sample size.

© 2025 Judson SD, Dowdy D. *JAMA Network Open*.

eTable 1. TriNetX Cohorts*

**Cohorts**	**Definition**	**Codes**	**Size**
WNF Adult ICD-10 2013-2024<br><br>(referred to as WNF 2013-2024)	Any adult patient with ICD-10 code indicating West Nile infection without encephalitis or neurologic manifestation from Jan 1st 2013-Dec 31st 2024	**A92.30**: West Nile virus infection, unspecified,<br>**A92.39** West Nile virus infection with other complications<br><br>**Cannot have:**<br>**A92.31**: West Nile virus infection with encephalitis<br>**A92.32**: West Nile virus infection with other neurologic manifestation	**1,328** (60/101 HCOs)
WNND Adult ICD-10 2013-2024<br><br>(WNND 2013-2024)	Any adult patient with ICD-10 code indicating WNV encephalitis or neurologic manifestation from Jan 1st 2013-Dec 31st 2024	**A92.31**: West Nile virus infection with encephalitis<br>**A92.32**: West Nile virus infection with other neurologic manifestation<br><br>**Cannot have:**<br>**A92.30**: West Nile virus infection, unspecified,<br>**A92.39** West Nile virus infection with other complications	**1,206** (57/101 HCOs)
WNV Adult ICD-10 2013-2024 deaths<br><br>(WNV & death 2013-2024)	Any adult patient with diagnosed West Nile infection who died from Jan 1st 2013-Dec 31st 2024	**A92.3**: West Nile virus infection,<br>**A92.30**: West Nile virus infection, unspecified<br>**A92.31**: West Nile virus infection with encephalitis<br>**A92.32**: West Nile virus infection with other neurologic manifestation<br>**A92.39**: West Nile virus infection with other complications<br><br>**AND Deceased**	**392** (44/101 HCOs)
WNV Adult ICD-10 2013-2024 non-deceased<br><br>(WNV & no death 2013-2024)	Any adult patient with diagnosed West Nile infection from Jan 1st 2013-Dec 31st 2024, excluding deceased	**A92.3**: West Nile virus infection,<br>**A92.30**: West Nile virus infection, unspecified<br>**A92.31**: West Nile virus infection with encephalitis<br>**A92.32**: West Nile virus infection with other neurologic manifestation<br>**A92.39**: West Nile virus infection with other complications<br><br>**Cannot have: Deceased**	**2,532** (65/101 HCOs)
WNV Adult all ICD-10 2013-2024<br><br>(WNV 2013-2024)	Any adult patient with diagnosed West Nile infection from Jan 1st 2013-Dec 31st 2024	**A92.3**: West Nile virus infection,<br>**A92.30**: West Nile virus infection, unspecified<br>**A92.31**: West Nile virus infection with encephalitis<br>**A92.32**: West Nile virus infection with other neurologic manifestation<br>**A92.39**: West Nile virus infection with other complications	**3,064** (65/101 HCOs)

*Cohort queries made on March 28th, 2025

© 2025 Judson SD, Dowdy D. *JAMA Network Open*.

eTable 2. Initial Cohort Characteristics Assessed

Covariate	TriNetX Criteria
Age	Age at index (WNV diagnosis)
Sex	Male, Female, Unknown
Ethnic group	Hispanic or Latino, Not Hispanic or Latino, Unknown
Race	White, Black or African American, Asian, Native Hawaiian or Other Pacific Islander, American Indian or Alaska Native, Other, Unknown
Malignant neoplasms of lymphoid, hematopoietic and related tissue (Hematologic malignant neoplasms)	C81-C96
Ischemic heart disease	I20-I25
Other forms of heart disease	I30-I5A
Diabetes	E08-E13
HIV	B20
CKD	N18
Liver Disease	K70-74
Alcohol related disorders	F10
Multiple Sclerosis	G35
Dementia	F03
Asthma	J45
COPD	J44
Essential (primary) hypertension	I10
Cerebrovascular disease	I60-163
Rheumatoid Arthritis	M06
Solid Organ Transplant	Z94
Antineoplastics	AN00
Immune suppressants	IM600 (includes calcineurin inhibitors, biologics)
Prednisone	8640
Tacrolimus	42316
Mycophenolate mofetil	68149

© 2025 Judson SD, Dowdy D. *JAMA Network Open*.

eTable 3. Baseline Characteristics of WNV and Death vs WNV and No Death 2013-2024 Cohorts

Covariate	Patients, No. (%) WNV & death (n = 368)	Patients, No. (%) WNV & no death (n = 2489)	[a]P-value
Age at diagnosis, mean (SD), y	69 (13)	56 (7)	<0.001
Sex			
Female	117 (32)	1099 (44)	<0.001
Male	243 (66)	1349 (54)	<0.001
Unknown	[b]≤10 (3)	41 (2)	0.15
Ethnicity			
Non-Hispanic or non-Latino	305 (83)	1892 (76)	0.004
Hispanic or Latino	23 (6)	198 (8)	0.25
Unknown	40 (11)	399 (16)	0.01
Race			
American Indian or Alaska Native	[b]≤10 (1)	[b]≤10 (1)	0.19
Asian	13 (4)	55 (2)	0.12
Black or African American	41 (11)	166 (7)	0.002
Native Hawaiian or Other Pacific Islander	[b]≤10 (1)	[b]≤10 (1)	0.19
[c]Other	[b]≤10 (3)	123 (5)	0.06
Unknown	29 (8)	204 (8)	0.83
White	270 (73)	1924 (77)	0.1
Hematologic malignant neoplasms	28 (8)	76 (3)	<0.001
Ischemic heart diseases	78 (21)	201 (8)	<0.001
Other forms of heart disease	148 (40)	464 (19)	<0.001
Diabetes	74 (20)	352 (14)	0.003
HIV	[b]≤10 (3)	21 (1)	0.001
Chronic kidney disease	70 (19)	170 (7)	<0.001
Diseases of the liver	36 (10)	140 (6)	0.002
Essential (primary) hypertension	112 (30)	745 (30)	0.84
Alcohol related disorders	17 (5)	86 (3)	0.26
Cerebrovascular diseases	70 (19)	213 (9)	<0.001
Chronic obstructive pulmonary disease	29 (8)	74 (3)	<0.001
Asthma	16 (4)	115 (5)	0.81
Multiple sclerosis	[b]≤10 (3)	36 (2)	0.07
Dementia	12 (3)	29 (1)	0.002
Other rheumatoid arthritis	11 (3)	47 (2)	0.16
Transplanted organ	17 (5)	55 (2)	0.006
Medication			
Immune suppressants (overall)	27 (7)	67 (3)	<0.001
Tacrolimus	13 (4)	42 (2)	0.02
Mycophenolate mofetil	16 (4)	30 (1)	<0.001
Antineoplastics	15 (4)	56 (2)	0.04
Prednisone	42 (11)	156 (6)	<0.001

[a]Continuous data were calculated using independent t-tests, and categorical data were calculated using χ^2 or Fisher's exact tests

[b]TriNetX patient counts of 10 or fewer were rounded up to 10 to maintain anonymity

[c]Included patients with race not matching the other categories above

© 2025 Judson SD, Dowdy D. *JAMA Network Open*.

Multivariable Cox Proportional Hazards Models: Mortality Outcomes

**eTable 4.** Mortality Within 90 Days		
**Covariate**	**AHR**	**95% Confidence Interval (P-value)**
WNND	2.58	1.58, 4.22 (< 0.001)
Male	0.86	0.56, 1.32 (0.49)
Age (per 10 years)	1.28	1.09, 1.51 (0.003)
Ischemic heart diseases	1.09	0.65, 1.83 (0.75)
Other forms of heart disease	3.94	2.40, 6.47 (< 0.001)
Chronic kidney disease	2.24	1.33, 3.75 (0.002)
Cerebrovascular diseases	1.89	1.17, 3.07 (0.01)
Chronic obstructive pulmonary disease	1.05	0.49, 2.24 (0.91)
Diabetes mellitus	0.73	0.43, 1.22 (0.23)
Hematologic malignancy	1.89	0.90, 3.95 (0.09)
Black or African American	0.87	0.39, 1.98 (0.75)
Immune Suppressants	1.10	0.50, 2.41 (0.82)
Unspecified dementia	0.50	0.12, 2.15 (0.35)
Diseases of the liver	2.14	1.18, 3.89 (0.01)
HIV	0.75	0.151, 3.757 (0.73)
Antineoplastics	1.66	0.57, 4.86 (0.35)
Prednisone	1.59	0.78, 3.24 (0.21)
Non-Hispanic or non-Latino	0.87	0.52, 1.45 (0.58)

Abbreviations: AHR, adjusted hazard ratio; WNND, West Nile neuroinvasive disease

© 2025 Judson SD, Dowdy D. *JAMA Network Open*.

**eTable 5.** Overall Mortality		
**Covariate**	**AHR**	**95% Confidence Interval (P-value)**
WNND	2.00	1.56, 2.57 (< 0.001)
Male	1.28	1.00, 1.65 (0.05)
Age (per 10 years)	1.51	1.37, 1.66 (< 0.001)
Ischemic heart diseases	1.36	1.00, 1.85 (0.05)
Other forms of heart disease	1.72	1.30, 2.26 (< 0.001)
Chronic kidney disease	1.99	1.45, 2.74 (< 0.001)
Cerebrovascular diseases	1.41	1.04, 1.91 (0.03)
Chronic obstructive pulmonary disease	1.76	1.16, 2.69 (0.008)
Diabetes mellitus	1.24	0.94, 1.65 (0.13)
Hematologic malignancy	1.62	1.01, 2.60 (0.05)
Black or African American	1.51	0.99, 2.29 (0.05)
Immune Suppressants	1.33	0.83, 2.13 (0.23)
Unspecified dementia	1.03	0.51, 2.08 (0.94)
Diseases of the liver	2.06	1.41, 3.00 (< 0.001)
HIV	0.69	0.25, 1.95 (0.49)
Antineoplastics	1.76	1.01, 3.05 (0.05)
Prednisone	1.86	1.23, 2.80 (0.003)
Non-Hispanic or non-Latino	1.27	0.92, 1.75 (0.15)

Abbreviations: AHR, adjusted hazard ratio; WNND, West Nile neuroinvasive disease

© 2025 Judson SD, Dowdy D. *JAMA Network Open*.

eFigure 1. Time-to-Event Analysis: WNND

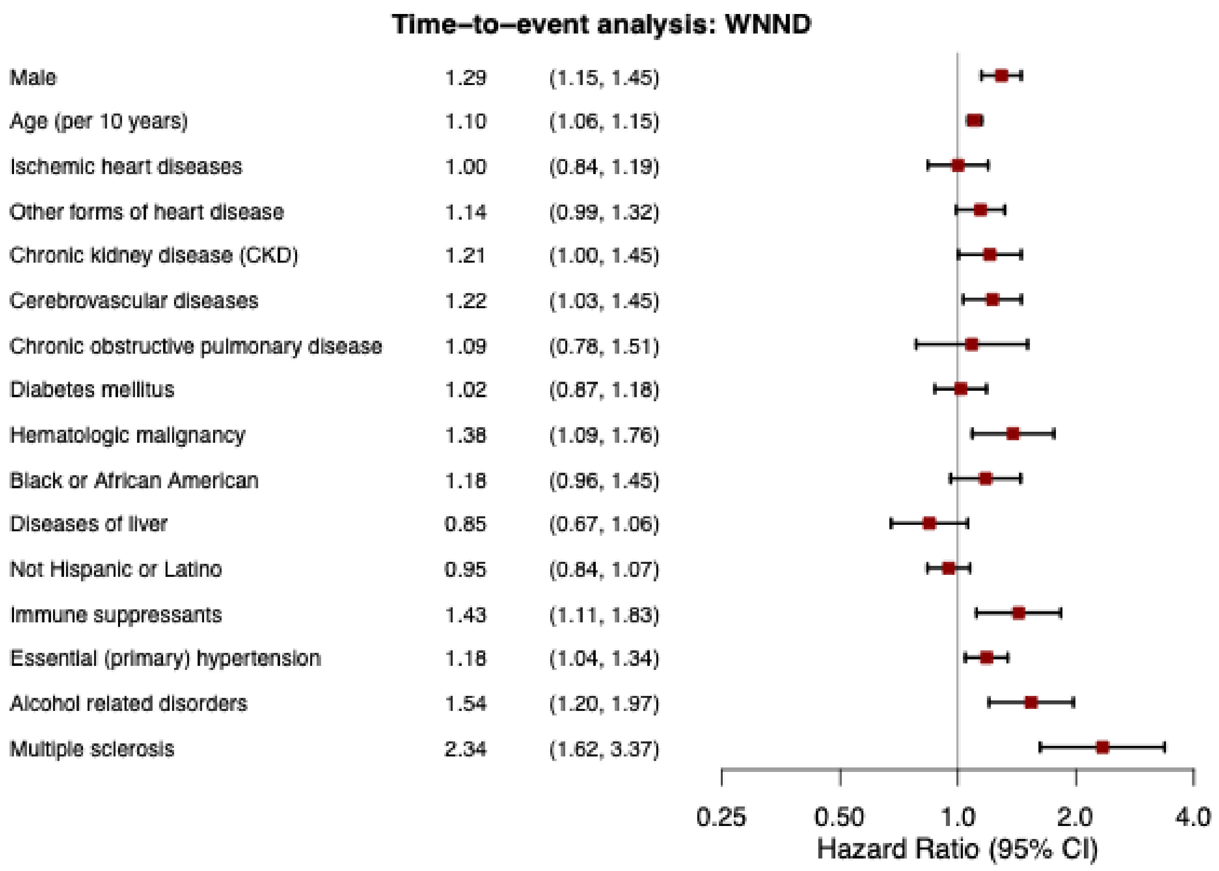

© 2025 Judson SD, Dowdy D. *JAMA Network Open*.

eFigure 2. Time-to-Event Analysis: 30-Day Mortality

Time–to–event analysis: 30 day mortality

WNND	2.49	(1.37, 4.52)
Male	0.65	(0.39, 1.08)
Age (per 10 years)	1.32	(1.07, 1.60)
Ischemic heart diseases	1.07	(0.57, 2.01)
Other forms of heart disease	5.50	(2.96, 10.23)
Chronic kidney disease (CKD)	2.08	(1.10, 3.93)
Cerebrovascular diseases	2.00	(1.14, 3.50)
Chronic obstructive pulmonary disease	1.50	(0.66, 3.42)
Diabetes mellitus	0.59	(0.31, 1.13)
Hematologic malignancy	1.55	(0.60, 4.04)
Black or African American	0.68	(0.22, 2.09)
Immune Suppressants	0.58	(0.17, 2.04)
Unspecified dementia	0.43	(0.06, 3.21)
Diseases of liver	1.95	(0.93, 4.08)
Human immunodeficiency virus [HIV] disease	0.57	(0.06, 5.31)
Antineoplastics	0.71	(0.09, 5.41)
Prednisone	1.22	(0.45, 3.32)
Not Hispanic or Latino	0.95	(0.51, 1.78)

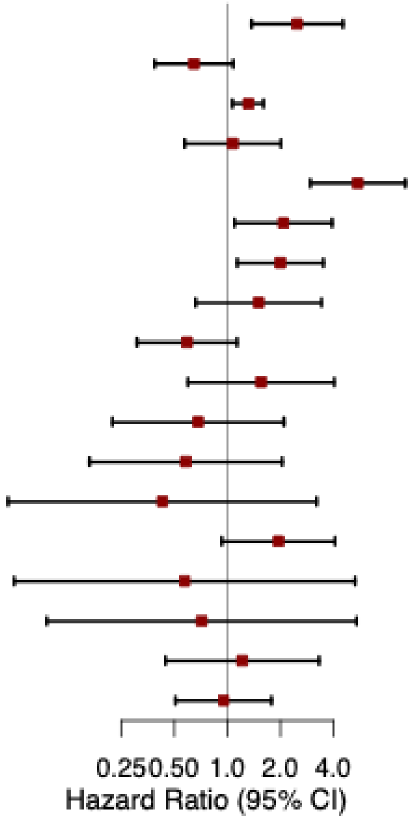

© 2025 Judson SD, Dowdy D. *JAMA Network Open*.

eFigure 3. Time-to-Event Analysis: 90-Day Mortality

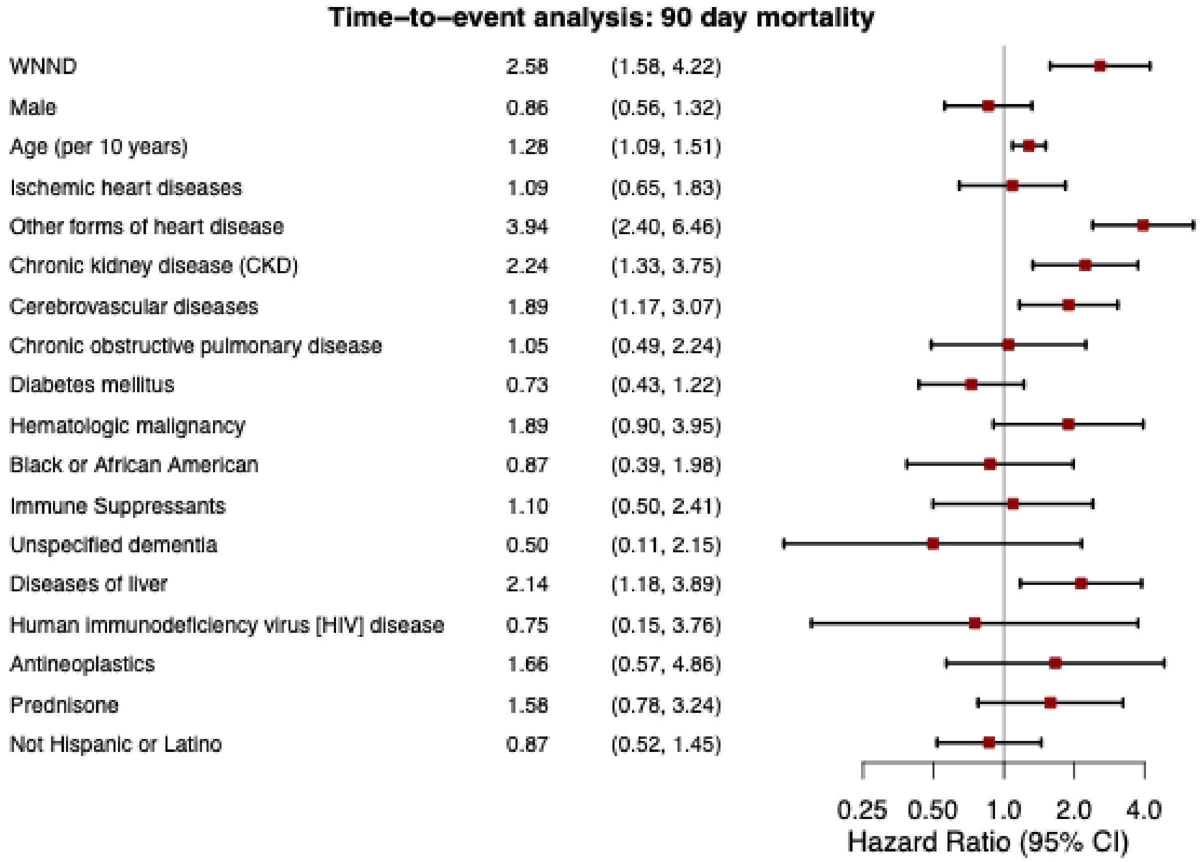

© 2025 Judson SD, Dowdy D. *JAMA Network Open*.

eFigure 4. Time-to-Event Analysis: Overall Mortality

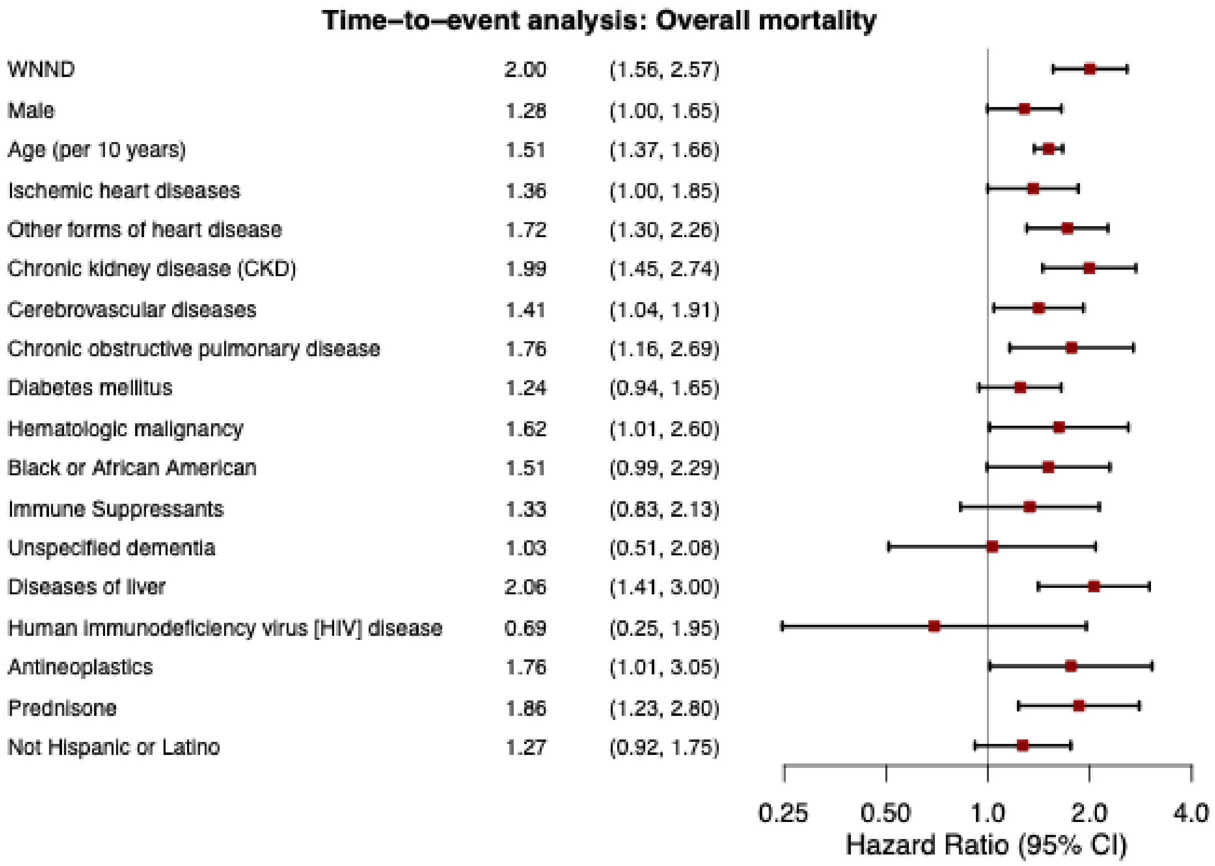

© 2025 Judson SD, Dowdy D. *JAMA Network Open*.
