# Supplementary material for: Risk Factors for West Nile Neuroinvasive Disease and Mortality in the US, 2013-2024
Source: JAMA Netw Open. 2025 Dec 10;8(12):e2548229. doi: 10.1001/jamanetworkopen.2025.48229 (PMC12696598; doi:10.1001/jamanetworkopen.2025.48229)
Supplement: Supplement 2. — Data Sharing Statement [file jamanetwopen-e2548229-s002.pdf]

Data Sharing Statement

Judson. Risk Factors for West Nile Neuroinvasive Disease and Mortality in the US, 2013-2024. *JAMA Netw Open*. Published December 10, 2025. doi:10.1001/jamanetworkopen.2025.48229

Data
Data available: No

Additional Information
Explanation for why data not available: Data for this study were accessed and analyzed using the TriNetX platform.
